# Acknowledgement to Referees

**DOI:** 10.1007/s13205-016-0551-2

**Published:** 2016-11-28

The Chief Editors and the Editorial Board thank the reviewers' listed below for their contribution and time consuming efforts in refereeing papers submitted to 3 Biotech in 2015. Their timely response and critical comments have not only assisted us in the selection of high-quality scientific work, but their critique is also appreciated by authors as it frequently assists them to improve the style and content of their publications.

Nayera A.M. Abdelwahed

María Abad-Grau

Naglaa Abdallah

Nadia AbdEl-Nasser

Tapan K. Adhya

Deepti Agrawal

Basir Ahmad

Ata Akcil

Mohd. Akhtar

Ayodele Alaiya

Sahal Al-Hajoj

Fatimah Alhamlan

Rabih Al-Kaysi

Sulaiman Al-Mayouf

Gundi Vijay Anand

Prof. Reshetilov Anatoly

Anees Ansari

Mohammad Ansari

Raheel Anwar

Abdolreza Ardeshirylajimi

Ehtasham Arif

Mohammad Asadollahi

Utku Avci

Md. Rabiul Awual

Yuxiang Bai

Chunyu Bai

N.K. Bainsala

M.D. Balakumaran

H.S. Balyan

Indrani Banerjee

Debdulal Banerjee

Hussaina Banu

Andrew Battle

P.D. Belur

Subhash Bhardwaj

Pankaj M. Bhatt

Durga Bhatt

Changhao Bi

S. Bindu

Rajib Biswas

Yannick Bomble

Abhijeet Borole

Craig Bunt

Siddhardha Busi Siddhardha

Tao Cai

Zhen Cai

Mingfeng Cao

Aurélie Cébron

Sonia Chadha

Susanta Chakraborty

Anindya Chanda

Nidhee Chaudhary

S.F. Chen

Jinchun Chen

Hairong Cheng

Joshua Chou

Kathrine Christensen

Hanna Dahm

P.K. Das

Ashis Das

Surajit Das

Shailesh Dave

Achlesh Daverey

Jan De Riek

Apurba Dey

D. Dhanasekaran

Nicolas Doucet

Joao Duarte

Kheireddine El-Boubbou

Nadia El-Gamal

Ahmed Elsharkawy

Sebnem Erenler

Leticia Estevinho

Thaddeus Ezeji

Anbin Ezhilan

E. Fernandez-Garcia

Daniela Gabor

Showkat Ganie

Yuhua Gao

Difeng Gao

Romain Gautier

K. Gawel-Beben

Xumeng Ge

Sveta Gerdes

Jan Geuns

M. Ghaedi

Parvatam Giridhar

Ananthan Gnanakkan

Venkateswara Gogineni

T. Gopalakrishna

Subramaniam Gopalakrishnan

Bhattiprolu Govinda Rao

Shipra Gupta

Dorin Gupta

Vandana Gupta

Vijai Kumar Gupta

Vijai Gupta

Baskar Gurunathan

Ashok Hadapad

Subhashish Haldar

Yiejun Han

Zhangying Hao

Vinayaka Hegde

Khan Hekmatyar

Cornelia Hooper

Shankar Hosmani

Zhiyong Huang

K. Hari Krishnan

Sundeep Jaglan

Rachna Jain

Hasnain Javed

R. Jayamadhuri

Venkatesh Jelli

Sujee Jeyapalina

Jean Jose

Chaitanya G. Joshi

Tarek Kabil

C.C.N. Khobragade

D. Kaladhar

Rajwant Kalia

Christos Kannas

Prasad Kaparaju

B. Kapdnis

Afsin Kaya

Kumaraswamy Kenchappa

R. Keerthi

Derek Kennedy

Ravi Kesari

Neda Keyhaninejad

Rafeiza Khan

Sardar Khan

Thida Win Khin

Moon-Soo Kim

Soo_Ki Kim

Uday Kishore

Timo Korhonen

Anil Kotasthane

Ankita Kothari

Fatemeh Kouhkan

Anagha Krishnan

Lakshminarasimhan Krishnaswamy

Ramesh Kuhad

Pankaj Kumar

Suresh Kumar

Dr. Narendra Kumar

Saravanah Kumar

Aaron Alfred Kwaasi

Park Kyeung

Jinwook Lee

Slawomir Lewicki

S.N. Lewis

Demao Li

Zhongyi Li

Jinshan Li

Y. Li

Zhenglong Li

Yuping Lin

S.J. Lin

Lifeng Liu

Tao Liu

Jin Liu

Jun Liu

Xiaowei Liu

H. Liu

Long Liu

José Pedro Lopes Faria

Xavier Louis

S. Lutsenko

Thomas Lutz

Hongwu Ma

Datta Madamwar

Rajat Mahajan

S. Dharne Mahesh

Hamid Maleki

Anushree Malik

K. Mallikarjuna

Nazim Mamedov

Jose Mancheno

Maegala Maniyam

Ashwini Mathur

Sayaji Mehetre

Boris Minaev

Pankaj Mishra

Giovanni Mita

M.K. Rajesh

Biplob Modak

Reda Moghaieb

Sanaa Mohamed

Maysa Moharam

Thanaa Mohdaly

Dejana Mokranjac

Hossain Mondal

Sukanta Mondal

Poulomi Mukherjee

Arup Kumar Mukherjee

Suprabhat Mukherjee

Suparna Mukherji

Akhtar Nadhman

Ambarish Nag

Manjunath Naik

Jyothisha Nair

Savithri Nambeesan

Bala Nambisan

Renu Nandakumar

Isali Nantes

Pradeep Negi

Helena Nevalainen

Sujogya Panda

Janmejay Pandey

Pranay Pankaj

Emmanuel Papamichael

M.V. Parakhia

Ramakrishnan Parthasarathi

Trupti Patel

Ranjana Pathania

Kishore Patil

Christina Payne

I. Peinado

Yanfeng Peng

Angelo Peralta

Carlo Piermarocchi

Onruthai Pinyakong

Vimal Prajapati

A.P. Pratap

Bharath Prithiviraj

T. Pullaiah

Hong Qing

Babak Rabiei

B. Rajasekhar Reddy

K. Raghavendra

Hamidur Rahaman

Sudhir Rai

Nishant Rai

Naushad Rais

S. Rajagopal

Alex Selvanayagam Rajangam

Bernard Rajeev SW

Vrinda Ramakrishnan

B.V. Raman

V. Rangaswamy

A.R. Rao

Rino Rappuoli

Mamoon Rashid

Arthi Rathinasabapathy

Gourav Rathore

Rajesh Raut

Prasun Ray

Manju Ray

Mehdi Razzaghi-abyaneh

Gopal Reddy

K. Rekha

Satyanarayana Rentala

Kedar Rokade

Mainak Roy

Seunghyun Ryu

A. Sabu

Jitendra Saini

S.S. Sandhu

Maddirevulla Sateesh

P. Suresh kumar

S. Satheesh

Kannusamy Sathyaseelan

Tulasi Satyanarayana

Indu Sawant

Walter Schmidt

Ivan Schuster

Samuel Seaver

G. Seghal Kiran

Flavio Seixas

Joseph Selvin

An Seong Soo A

Hanan Shaaban

Shafinaz Shahir

Sitansh Sharma

Birinchi Sharma

Nitya Sharma

R. Shashidhar

Ing-Lung Shih

P. Shilpkar

Wenqing Shui

Pratyoosh Shukla

Swapna Simon

Randhir Singh

Abhishek Singh

Kusum Solanki

Andong Song

Cunjiang Song

Hui Song

Maria Soto

Valentina Spanic

Janet Sprent

Bharati Srinivasan

S. Sriram

Manoj Srivastava

Alexander Steinbüchel

M. Subhosh Chandra

Mayavan Subramanian

Kondeti Subramanyam

Rajeev Sukumaran

Faheem Sultan Mohammad

Geeta Sumbali

Yuanxia Sun

P.N. Sunilkumar

Laura A. Svetaz

Tamer Tamer

Li Tan

Geok Hun Tan

Shuangyan Tang

Fei Tao

Hamsa Tayeb

Valentino Te’o

Chao-guang Tian

Adeline TIng

Nathan Tintle

Joe Tiralongo

Montserrat Tobajas

M. Tohidfar

Irena Trbojević Akmačić

Minh Tri

Timir Tripathy

Ran Tu

P. Uma Maheswari Devi

Naryana Upadhyaya

S.V.N. Vijayendra

O.P. Verma

Salwa Wahsh

Dongping Wang

Zhen Wang

Yi Wang

Haiyan Wang

Chonglong Wang

Thomas West

Qingyu Wu

Yi-Rui Wu

Qiaqing Wu

Haixia Xiao

Jianmin Xing

Zhenghong Xu

Zhuofei Xu

S. Xu

Vinod Yadav

John Yarbrough

Göksungur Yekta

Dr. Sailu Yellaboina

Rachel Yesudasan

Doctor Xiaoxing Yin

Yanbin Yin

Bo Yu

Xiaochen Yu

Yongbo Yuan

Youxi Yuan

Haggag Zain

Lila Zarei

Y.L. Zhang

Yanping Zhang

Guimin Zhang

Dongyuan Zhang

Ling Zhang

M.W. Zhang

Y. Zhang

Yifeng Zhang

Dawei Zhang

Dong Zhang

Q. Zhang

Yingying Zheng

Che Zhen-ming

Cheng Zhou

Kun Zhu

